# Visual annotation display (VLAD): a tool for finding functional themes in lists of genes

**DOI:** 10.1007/s00335-015-9570-2

**Published:** 2015-06-06

**Authors:** Joel E. Richardson, Carol J. Bult

**Affiliations:** Mouse Genome Informatics (MGI) Database Consortium, The Jackson Laboratory, 600 Main Street, Bar Harbor, ME 04609 USA

## Abstract

Experiments that employ genome scale technology platforms frequently result in lists of tens to thousands of genes with potential significance to a specific biological process or disease. Searching for biologically relevant connections among the genes or gene products in these lists is a common data analysis task. We have implemented a software application for uncovering functional themes in sets of genes based on their annotations to bio-ontologies, such as the gene ontology and the mammalian phenotype ontology. The application, called VisuaL Annotation Display (VLAD), performs a statistical analysis to test for the enrichment of ontology terms in a set of genes submitted by a researcher. The results for each analysis using VLAD includes a table of ontology terms, sorted in decreasing order of significance. Each row contains the term, statistics such as the number of annotated terms, the *p* value, etc., and the symbols of annotated genes. An accompanying graphical display shows portions of the ontology hierarchy, where node sizes are scaled based on *p* values. Although numerous ontology term enrichment programs already exist, VLAD is unique in that it allows users to upload their own annotation files and ontologies for customized term enrichment analyses, supports the analysis of multiple gene sets at once, provides interfaces to customize graphical output, and is tightly integrated with functional and biological details about mouse genes in the Mouse Genome Informatics (MGI) database. VLAD is available as a web-based application from the MGI web site (http://proto.informatics.jax.org/prototypes/vlad/).

**Electronic supplementary material:**

The online version of this article (doi:10.1007/s00335-015-9570-2) contains supplementary material, which is available to authorized users.

## Introduction

One of the challenges facing biologists in the era of genome scale science is to glean biological meaning from large experimental datasets such as those generated by microarray, RNA Seq, ChIP (chromatin immunoprecipitation) Seq, genome wide copy number variation (CNV) analysis, and exome sequencing. The development of biomedical ontologies such as the Gene Ontology (GO) (Ashburner et al. 2000; Gene Ontology 2015) and annotated gene sets (Subramanian et al. 2005) have been essential for mining functional properties of genes from large-scale datasets.

Numerous software tools that use curated annotations and ontologies for extracting functional information from gene sets have been developed over the years including GO::TermFinder (Boyle et al. 2004), DAVID (da Huang et al. 2009), BiNGO (Maere et al. 2005), AmiGO (Carbon et al. 2009), GoMiner (Zeeberg et al. 2003), and WebGestalt (Wang et al. 2013). In general, these programs are designed to analyze gene sets that show statistically significant patterns of gene expression, variation, etc. Other gene set analysis methods such as gene set enrichment analysis (GSEA) (Subramanian et al. 2005), parametric analysis of gene set enrichment (PAGE) (Kim and Volsky 2005), and generally applicable gene set enrichment (GAGE) (Luo et al. 2009) allow for the analysis of all genes in global transcriptomics studies. These methods were developed to address the issue that not all meaningful gene expression changes rise to the level of statistical significance. Both the “cutoff -based” and “cutoff-free” methods (Luo et al. 2009) rely on comparisons of experimental gene sets to annotated gene sets and ontologies to facilitate data interpretation.

We describe here a web-based application called VLAD (VisuaL Annotation Display) for finding functional themes in sets of genes based on their ontology term annotations. VLAD uses the hypergeometric test for determining significance and is appropriate for the analysis of gene sets that are generated by “cutoff-based” statistical analyses methods. VLAD is highly configurable; there are many parameters that can be set by users that control input, data processing, and output. A unique feature of the software relative to existing term enrichment tools is that it is not limited to the two native ontologies in the system: the Gene Ontology (Ashburner et al. 2000) and the Mammalian Phenotype Ontology (Smith and Eppig 2012); rather, VLAD can compare lists of genes to any structured vocabulary that is in the standard open biological and biomedical ontologies (OBO) format (http://www.obofoundry.org) (Smith et al. 2007) and for which there is a file of gene-to-annotation-term associations in the GO Annotation Format (GAF; http://geneontology.org/page/go-annotation-file-gaf-format-10). VLAD also provides users with a level of control over the graphical display of results that is not available in other similar analysis sites. VLAD is available as a web-based application from the Mouse Genome Informatics (MGI) web site (http://proto.informatics.jax.org/prototypes/vlad/).

## Materials and methods

### Data sources

To illustrate the functionality of VLAD, we analyzed genes from a previously published study that described the genome wide gene expression patterns across key developmental stages of normal mouse diaphragm (Russell et al. 2012). In this study, the investigators used time-series analysis (Ernst and Bar-Joseph 2006) of microarray-based expression data to identify over 650 genes whose expression levels increased significantly between embryonic day 11.5 and embryonic day 16.5 and over 360 genes whose expression levels decreased significantly over this same time period.

To demonstrate the extensibility of VLAD to user-provided ontologies, an OBO ontology of mouse biochemical pathways (mousecyc_obo.txt) and a corresponding set of annotations in GAF format (mousecyc_gaf.txt) from the curated MouseCyc database (Evsikov et al. 2009) were downloaded from the MouseCyc project ftp site (ftp://informatics.jax.org/pub/curatorwork/MouseCycDB/) and chosen as the basis for a custom term enrichment analysis using the Annotation Data Set options on the VLAD homepage. The mouse diaphragm gene lists, OBO ontology, and GAF files are available as supplemental data and from the following ftp site: ftp://informatics.jax.org/pub/supplemental/MammGenome2015.

### Running VLAD

VLAD is preconfigured to work with either gene-function annotations from MGI using the GO and/or gene-phenotype annotations from MGI using the Mammalian Phenotype (MP) Ontology. The GO and MP annotations used by VLAD are updated weekly. A user may also upload a different ontology (a file in OBO format) and corresponding annotation dataset (a file in GAF format) to perform custom enrichments. Users may also use the built in MP and GO ontologies but supply their own gene-to-ontology term annotations.

To run an analysis with VLAD users submit one or more test sets of gene symbols or accession identifiers for the laboratory mouse. By default, the test set of genes is compared to the annotations for all genes in the mouse reference genome to determine the likelihood that the annotation terms associated with the test set would occur by chance. Alternatively, users can submit a custom list of genes to which their test set should be compared. This option may be preferable for the analysis of lists of genes from studies that use a targeted set of genes for analysis; the distribution of annotations for genes for such targeted studies may be quite different than the annotations for the genome as a whole. Annotations from all sources of evidence are included in the analysis by default. The user has the option of limiting the analysis to only those annotations derived from specific classes of evidence. For example, it may be desirable to limit analyses to only those annotations derived from direct experimental assays as opposed to those inferred from sequence similarity or homology. For custom enrichment analyses, users can include their own evidence codes in the input GAF file. These user-supplied codes can be specified in the evidence code parameter settings of VLAD to exclude specific sets of annotations from the enrichment analysis.

A unique feature of VLAD is the support for the analysis of multiple gene sets at a time. For example, the up-regulated and down-regulated gene sets from a transcriptomics experiment can be analyzed at the same time to evaluate the biological consequence of gene expression changes from the perspective of biological function. Each gene set is analyzed independently, and the results are shown in a combined display designed to reveal enrichment differences between the sets.

### Calculating statistical significance of annotations for a gene set

Suppose out of a list of 100 genes up-regulated in a disease sample relative to normal, 40 are associated with mortality/aging phenotypes. Is 40 % significant? What would we expect to see if we simply picked 100 genes at random? Like many other ontology term enrichment tools, VLAD calculates significance based on the hypergeometric distribution. For every term, *t*, in the ontology, VLAD computes a *p* value, *p*_*t*_(*k*, *n*, *K*, *N*), where *k* is the number of genes in the query set annotated to *t* or its descendants, *n* is the size of the query set, *K* is the total number of genes in the database annotated to *t* or its descendants, and *N* is the total number of annotated genes. The results are sorted with the terms of highest significance at the top. The default analysis in VLAD is for term enrichment where the *p* value is the probability of drawing *at leastk* successes in *n* tries given a population of *K* out of *N*. VLAD also offers the option of performing a depletion analysis where *p* is the probability of drawing *at mostk* successes in *n* tries.

One issue that VLAD and similar tools must deal with is the multiple testing problem (Noble 2009), which in this case means that the reported *p* values are inflated simply because we are calculating them for so many terms (i.e., doing many tests). To account for multiple testing in VLAD an additional statistic, the *q* value is calculated, which is based on the concept of the positive false discovery rate (pFDR) (Storey 2002). The *q* value is the proportion of false positives when a given group of tests is called significant and is easily computed from the ordered *p* values. In terms of the results generated by VLAD, the *q* value in row *i*, *q*_i_, is interpreted as the rate of false positives if we were to consider all terms in rows 0…i to be significant.

### VLAD output

The output from the VLAD program includes both graphical and tabular representations of the ontology terms associated with the user-supplied gene list (i.e., the query set) and the calculated significance scores. The tabular display shows the detailed results, i.e., all the ontology terms, their scores, statistics, and associated genes, sorted in order of decreasing significance. The graphical display provides a high level visual summary of the most significant terms from table. Each node in the graph corresponds to a term and node sizes are scaled by term significance. VLAD uses GraphViz (Gansner and North 1999) (http://www.graphviz.org/) for graph layout and visualization. The nodes in the graph and the rows in the tabular view are cross-linked so the user can easily move between the two kinds of display. The results of a gene set analyses in VLAD can be downloaded and saved to a user’s local disk. VLAD also provides the option of downloading results as an Excel spreadsheet or tab-delimited file.

VLAD provides numerous options allowing the user to customize color, image size, and nodes to display. Even for small sets of genes, the number of associated ontology terms, and hence, the size of the resulting image, may be large. VLAD provides adjustable parameters that allow the user to limit the number and reduce the size of the nodes drawn in the image. The “limit nodes” option allows the user to display only those nodes that meet specific scoring criteria. The “cull interior nodes” option allows the user to further reduce the size of the graphic by omitting many uninformative interior nodes from the display. This option is “on” by default. The root node in the ontology is always included regardless of the settings because it is visually important and helps establish context for the user.

**Fig. 1:**
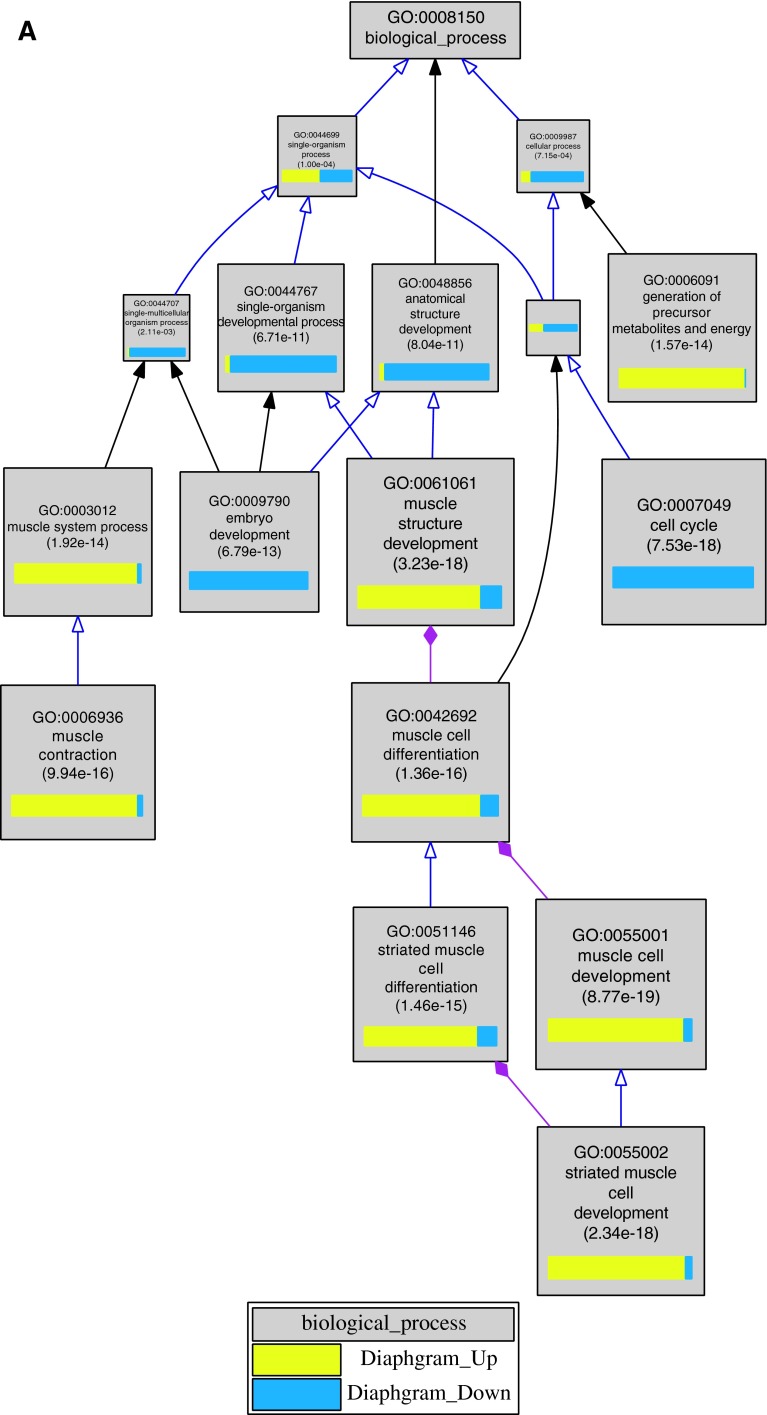

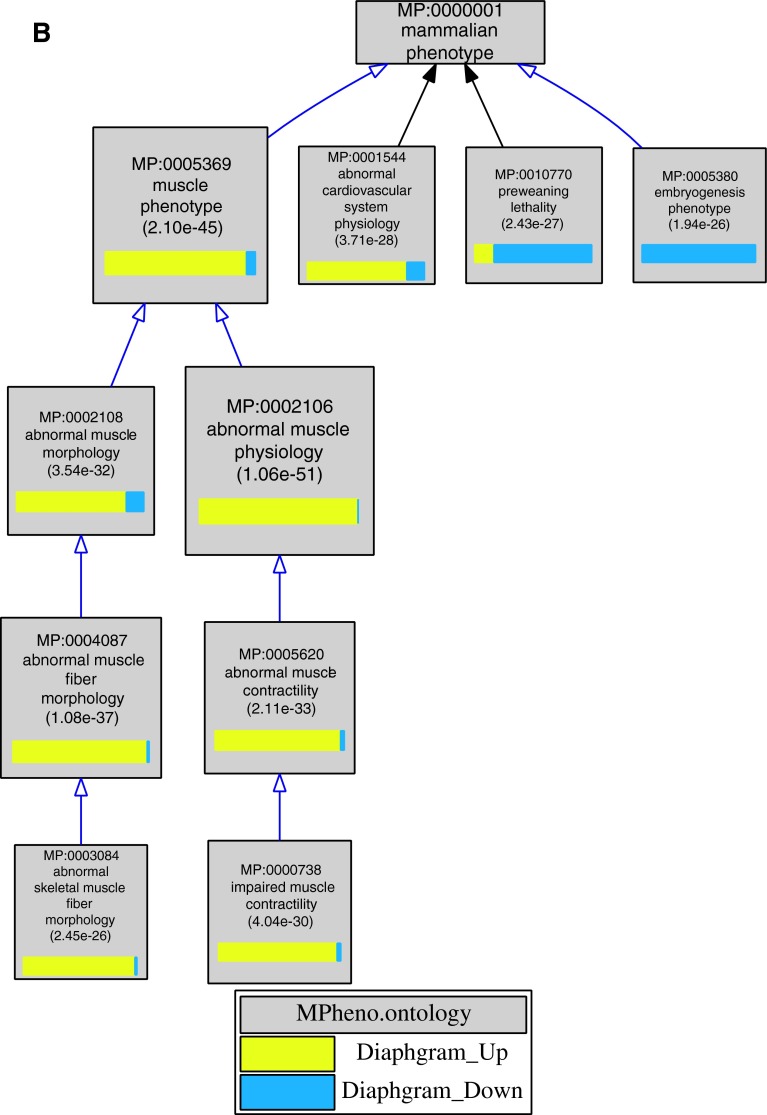
Example of the graphical output from VLAD for the analysis of genes whose expression levels go up (*yellow*) and down (*blue*) over time during normal diaphragm development in the laboratory mouse (Russell et al. 2012) for GO term enrichment (*panel A*) and Mammalian Phenotype Ontology term enrichment (*panel B*). The size of a node reflects its score: the larger the node, the greater its statistical significance. Because there are two gene sets, VLAD calculates two significance scores for each node; the minimum (most significant) score is used to draw the node. The *colored bars* in each node indicate the relative sizes of the two scores. The values displayed in each node are, in order, the term identifier, the term name, and the significance score (*p* value). The* lines* (edges) connecting the nodes in the* graph* represent the relationships between the terms. A *purple line* *with a solid, diamond*-*shaped tip* represents a “part-of” relationship between terms; a *blue line with a hollow arrow tip* represents an “is-a” relationship between the terms. A *black line with a solid arrow tip* indicates that several nodes in a multi-step path are not being displayed in order to simplify the graphic. The user can configure the* colors *used (node, datasets, background) the number of nodes displayed, and the overall size of the image generated

When analyzing multiple gene sets at once, the user may assign colors to the sets, which then appear in both displays to help with comparison (Fig. 1). The color and style of the edges that connect the nodes represent the relationship between the terms (i.e., is-a, part-of, positively regulates, negatively regulates).

## Results

The lists of up- and down-regulated genes from a published mouse diaphragm development study (Russell et al. 2012) were analyzed for term enrichment for both the gene ontology and Mammalian Phenotype Ontology using VLAD. The graphical output for both of these analyses is shown in Fig. 1a, b. The results of the VLAD analysis demonstrate that the genes that decline over time during diaphragm development are primarily involved with general mammalian organogenesis and systems development, while genes that increase are associated with muscle development and contractility (Fig. 1a). Likewise, the down-regulated genes are enriched in phenotypes associated with abnormal survival, while the up-regulated gene set is associated with abnormal muscle phenotypes (Fig. 1b). The option in VLAD to display the enrichment results for both sets of genes simultaneously helps to highlight the biological and functional differences represented in the two sets of genes.

**Fig. 2:**
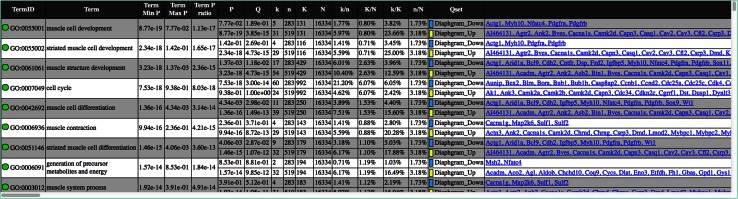
Screenshot of VLAD’s tabular output for GO terms associated with the graphical output displayed in Fig. 1, panel A. Two gene sets were analyzed at once, so there are two sets of statistics for each ontology term. A term’s overall score is the minimum its two *p* values. The GO terms are hyperlinked to the AmiGO database at Stanford University. In addition,* each row* is linked (via the *green circle*) to its node in the graphical display, and vice versa, for easy navigation between the two. For mouse genes, the* symbols* are hypertext linked to the gene detail information from the Mouse Genome Informatics (MGI) database

The tabular output from VLAD associated with Fig. 1 is shown in Fig. 2. Each row corresponds to one ontology term and contains all the summary statistics, significance scores, and list of annotated genes. The table is sorted by decreasing significance. Each node in the graphical display is hyperlinked to its row in the table and vice versa. The GO terms in the tabular output (Fig. 2) are hyperlinked to the AmiGO Browser (Carbon et al. 2009) at Stanford (http://www.godatabase.org/cgi-bin/amigo/go.cgi). For VLAD analyses that involve mouse genes, each gene in the tabular output (Fig. 2) is hyperlinked to extensive biological information about the gene available from the Mouse Genome Informatics (MGI) (Eppig et al. 2015) database (http://www.informatics.jax.org).

**Fig. 3:**
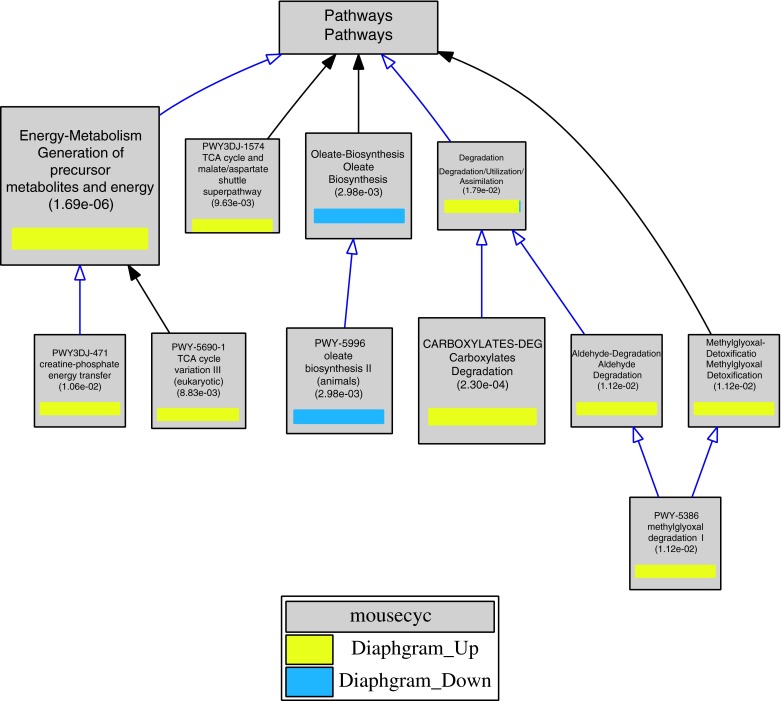
Screenshot of the graphical output from VLAD for a custom term enrichment analysis using a user-provided ontology of biochemical pathways from the MouseCyc database and an associated gene-to-pathway term annotation file

The results of a custom term enrichment analysis based on a user-provided ontology of biochemical pathway terms from the MouseCyc database is shown in Fig. 3. The results of this analysis demonstrates that genes that are up-regulated during normal diaphragm development are enriched in pathways associated with energy metabolism and detoxification, while the genes whose expression levels decrease over time are associated with fatty acid biosynthesis pathways.

## Discussion

VLAD is a highly configurable web-based exploratory data analysis tool for the term enrichment analysis. Compared to web-based software tools with comparable functionality, VLAD is unique in the degree to which it can be adapted easily to use any ontology and annotation combination. In its current implementation, VLAD is designed to work best for term enrichment/depletion analysis for the laboratory mouse. Planned enhancements to the software will include support for more species, specifically for human gene sets. Future versions of VLAD will include additional algorithms for term enrichment (e.g., (Bauer et al. 2010; Glass and Girvan 2014; Prufer et al. 2007) and allow users to share ontology and annotation files they use for custom term enrichment analyses.

VLAD is available as a web-based application from the Mouse Genome Informatics (MGI) web site at the following URL: http://proto.informatics.jax.org/prototypes/vlad/. User documentation for VLAD is provided online from the VLAD home page.

## Electronic supplementary material

Supplementary material 1 (XLSX 24 kb)

Supplementary material 2 (XLSX 38 kb)

Supplementary material 3 (TXT 93 kb)

Supplementary material 4 (TXT 205 kb)

## References

[CR1] Ashburner M, Ball CA, Blake JA, Botstein D, Butler H, Cherry JM, Davis AP, Dolinski K, Dwight SS, Eppig JT, Harris MA, Hill DP, Issel-Tarver L, Kasarskis A, Lewis S, Matese JC, Richardson JE, Ringwald M, Rubin GM, Sherlock G (2000). Gene ontology: tool for the unification of biology. The Gene Ontology Consortium. Nat Genet.

[CR2] Bauer S, Gagneur J, Robinson PN (2010). GOing Bayesian: model-based gene set analysis of genome-scale data. Nucleic Acids Res.

[CR3] Boyle EI, Weng S, Gollub J, Jin H, Botstein D, Cherry JM, Sherlock G (2004). GO::termFinder–open source software for accessing Gene Ontology information and finding significantly enriched Gene Ontology terms associated with a list of genes. Bioinformatics.

[CR4] Carbon S, Ireland A, Mungall CJ, Shu S, Marshall B, Lewis S, Ami GOH, Web Presence Working G (2009). AmiGO: online access to ontology and annotation data. Bioinformatics.

[CR5] da Huang W, Sherman BT, Lempicki RA (2009). Systematic and integrative analysis of large gene lists using DAVID bioinformatics resources. Nat Protoc.

[CR6] Eppig JT, Blake JA, Bult CJ, Kadin JA, Richardson JE, Mouse Genome Database G (2015). The Mouse Genome Database (MGD): facilitating mouse as a model for human biology and disease. Nucleic Acids Res.

[CR7] Ernst J, Bar-Joseph Z (2006). STEM: a tool for the analysis of short time series gene expression data. BMC Bioinform.

[CR8] Evsikov AV, Dolan ME, Genrich MP, Patek E, Bult CJ (2009). MouseCyc: a curated biochemical pathways database for the laboratory mouse. Genome Biol.

[CR9] Gansner E, North S (1999). An open graph visualization system and its applications to software engineering. Softw Pract Exp.

[CR10] Gene Ontology C (2015). Gene Ontology Consortium: going forward. Nucleic Acids Res.

[CR11] Glass K, Girvan M (2014). Annotation enrichment analysis: an alternative method for evaluating the functional properties of gene sets. Sci Rep.

[CR12] Kim SY, Volsky DJ (2005). PAGE: parametric analysis of gene set enrichment. BMC Bioinform.

[CR13] Luo W, Friedman MS, Shedden K, Hankenson KD, Woolf PJ (2009). GAGE: generally applicable gene set enrichment for pathway analysis. BMC Bioinform.

[CR14] Maere S, Heymans K, Kuiper M (2005). BiNGO: a Cytoscape plugin to assess overrepresentation of gene ontology categories in biological networks. Bioinformatics.

[CR15] Noble WS (2009). How does multiple testing correction work?. Nat Biotechnol.

[CR16] Prufer K, Muetzel B, Do HH, Weiss G, Khaitovich P, Rahm E, Paabo S, Lachmann M, Enard W (2007). FUNC: a package for detecting significant associations between gene sets and ontological annotations. BMC Bioinform.

[CR17] Russell MK, Longoni M, Wells J, Maalouf FI, Tracy AA, Loscertales M, Ackerman KG, Pober BR, Lage K, Bult CJ, Donahoe PK (2012). Congenital diaphragmatic hernia candidate genes derived from embryonic transcriptomes. Proc Natl Acad Sci USA.

[CR18] Smith CL, Eppig JT (2012). The Mammalian Phenotype Ontology as a unifying standard for experimental and high-throughput phenotyping data. Mamm Genome.

[CR19] Smith B, Ashburner M, Rosse C, Bard J, Bug W, Ceusters W, Goldberg LJ, Eilbeck K, Ireland A, Mungall CJ, Consortium OBI, Leontis N, Rocca-Serra P, Ruttenberg A, Sansone SA, Scheuermann RH, Shah N, Whetzel PL, Lewis S (2007). The OBO Foundry: coordinated evolution of ontologies to support biomedical data integration. Nature Biotechnol.

[CR20] Storey JD (2002). A direct approach to false discovery rates. J Roy Stat Soc B.

[CR21] Subramanian A, Tamayo P, Mootha VK, Mukherjee S, Ebert BL, Gillette MA, Paulovich A, Pomeroy SL, Golub TR, Lander ES, Mesirov JP (2005). Gene set enrichment analysis: a knowledge-based approach for interpreting genome-wide expression profiles. Proc Natl Acad Sci USA.

[CR22] Wang J, Duncan D, Shi Z, Zhang B (2013). WEB-based GEne SeT AnaLysis Toolkit (WebGestalt): update 2013. Nucleic Acids Res.

[CR23] Zeeberg BR, Feng W, Wang G, Wang MD, Fojo AT, Sunshine M, Narasimhan S, Kane DW, Reinhold WC, Lababidi S, Bussey KJ, Riss J, Barrett JC, Weinstein JN (2003). GoMiner: a resource for biological interpretation of genomic and proteomic data. Genome Biol.

